# Molecular mechanisms of microglia- and astrocyte-driven neurorestoration triggered by application of electromagnetic fields

**DOI:** 10.3325/cmj.2019.60.127

**Published:** 2019-04

**Authors:** Jasmina Isaković, Dunja Gorup, Dinko Mitrečić

**Affiliations:** 1 Omnion Research International, Zagreb, Croatia; 2 Department of Histology and Embryology, University of Zagreb School of Medicine, Zagreb, Croatia

## Abstract

**Aim:**

To propose potential mechanisms of action of electromagnetic fields (EMF) on astrocytes and microglia and to elucidate the role of heat shock proteins (HSP), adenosine triphosphate (ATP), calcium ions (Ca^2+^), and hypoxia-inducible factor 1α (HIF1α) in neurorestoration following the application of EMF.

**Methods:**

We reviewed the existing studies within the public domain and cross-evaluated their results in order to conclude on the molecular mechanisms of microglia-astrocyte crosstalk at work during EMF treatment.

**Results:**

The existing studies suggest that EMF induces the increase of HSP70 expression and inhibition of HIF1α, thus decreasing inflammation and allowing the microglia-astrocyte crosstalk to initiate the formation of a glial scar within the central nervous system. Furthermore, by potentially up-regulating A2A and A3 adenosine receptors, EMF increases cAMP accumulation from astrocytes and reduces the expression of inflammatory cytokines TNF α and IL-8, thus initiating neurorestoration.

**Conclusion:**

The microglia-astrocyte crosstalk during EMF treatment is crucial for the initiation of neurorestoration. Elucidating the exact mechanisms of EMF actions upon microglia and astrocytes, and its role in neurorestoration, could be a key step in further research of the therapeutic potential of EMFs in various neurological disorders.

## INTRODUCTION

### Neurodegeneration, neuroinflammation, and electromagnetic fields

Neurodegeneration is an umbrella term used to describe various processes that lead to a loss of structure or function of neurons, including their death. Considerable efforts have been directed at demystifying the role of inflammatory processes in neurodegeneration, and such expanded understanding could be helpful in devising novel therapeutic approaches targeting the neuroimmune system to initiate neurorestoration.

Neuroinflammation is a biological response of body tissues to harmful stimuli involving immune cells, blood vessels, and molecular mediators within the central nervous system (CNS). It includes attraction, migration, and accumulation of cells, which support a cascade of events participating in tissue damage and regeneration. In various CNS pathologies, the inflammatory response includes the activation of microglia and astrocytes.

Since it has been shown that electromagnetic fields (EMFs) are effective in reducing the pain of various etiologies through modification of innate immunity (1-4), it has been hypothesized that application of EMFs may either slow down neurodegeneration or decrease the neuroinflammatory processes within the central or peripheral nervous system (PNS) (5,6). This approach could help to initiate neurorestoration. Not only does this new method appear to be non-invasive, but it is also more cost-effective and safer than drugs and surgical procedures. Although multiple reviews aim to elucidate the molecular mechanisms behind the action of EMFs within the CNS (4,7-11), very little is still known about the cause of neurorestoration occurring after application of EMF treatment.

Treatment of autoimmune diseases, as well as general boosting of the immune system, has so far been performed by using drugs (12-16) or nanotechnology (17-21). The most recent trend in medical research aims to use EMFs to fine-tune the immune response through modulation of cell membrane charges, ion flow, and cell mobility, be it in the innate immune response within the CNS or the rest of the body (6,22-28).

Multiple studies have investigated the effects of EMFs on innate immunity and various pathogens, aiming to delineate the activation of molecular pathways that lead to success in EMF therapies (4,29,30). As the most abundant cells within the nervous system, astrocytes and microglia are the crucial players of the neuroimmune system. While microglia function as the first stage of defense against foreign pathogens, astrocytes are involved in repair and regeneration of the injured tissue. Microglia also regulate the innate immune function of astrocytes, thus determining their neuroprotective or neurotoxic function. In turn, astrocytes secrete molecules that trigger microglial activation and regulate microglial phenotypes and function by impacting their motility and phagocytosis.

Multiple studies investigated the impact of EMF on astrocyte and microglia function, yielding contrasting results, both *in vivo* and *in vitro* (31-38). This article aims to critically review the literature within the field, including both *in vivo* and *in vitro* studies, with the purpose of clarifying the molecular mechanisms at work, specifically when it comes to the crosstalk between astrocytes and microglia. Therefore, this review focuses on three main molecular targets – ATP, HSP, and HIF1α – which have been shown to be influenced by EMF the most in different cell types and tissues. The same molecular targets are potential candidates for further research on EMF’s influence on astrocytes and microglia and their role in assisted neurorestoration (1,31,34,36-38).

### EMF in the brain tissue: mechanisms of administration and action

Cell membrane potential and ion balance are maintained through the exchange of mainly sodium, potassium, and calcium ions, and are deranged in different pathological conditions. Both external and internal EMFs have the ability not only to modify charge distribution through directing the movement of ions within the cells and the extracellular space, but also to impact the opening and closing of some voltage gated calcium channels. This is why they have recently attracted an increasing interest as a form of therapy which may initiate tissue regeneration and restoration – this also includes the innate electromagnetic fields generated around neurons discussed in our previous article (6). Thus, the brain has entered the spotlight of EMF therapy, which can target either the innate immune cells within the CNS, the cells that govern the initiation of the immune response (such as microglia and astrocytes), or other cell types within the brain.

Currently, EMF therapy is most commonly being administered in the form of pulsed electromagnetic fields (PEMF), separated into low field magnetic stimulation (LFMS) or extremely low frequency magnetic fields (ELF-MF), extremely low frequency electromagnetic fields (ELF-EMF), tumor treating fields (TTF), and deep brain stimulation (DBS). Mechanisms of DBS action are well described, and this surgical procedure is successfully used in medical practice for treatment of disorders such as Parkinson’s, essential tremors, and epilepsy (39-41). PEMF, on the other hand, is a non-surgical procedure and has been proved to be useful for fracture healing (42). Electroencephalograms (EEG) and magnetoencephalograms (MEG), which have only been used in diagnostics, could also, in their current or modified form, be used as a form of EMF therapy as they influence the behavior of the cells within the CNS.

### Astrocyte and microglia crosstalk

*Molecular mechanisms of communication between astrocytes and microglia*. By being the primary immune cells within the CNS, microglia are involved in various neuropathological conditions and, together with astrocytes, help the CNS recover from stress and injury (43). Because of their autocrine feedback and bidirectional conversation during the modulation of CNS injury, the crosstalk between microglia and astrocytes has emerged into the forefront of glial research and EMF therapy used to initiate neurorestoration. At the center of this reciprocal modulation is the microglial regulation of the innate immune functions of astrocytes, determining their function, which can be neuroprotective or neurotoxic (43). Activated earlier than astrocytes, microglia secrete NADPH oxidase-derived H_2_O_2_ (44), interleukin 1 alpha (IL-1α), tumor necrosis factor alpha (TNFα), and complement component 1q (C1q) (45) to regulate astrocytic activation and initiate A1 reactive astrocytosis – the main destructive pathway of astrocytic activity. This all makes microglia the primary targets for EMF therapy of the CNS.

Although astrocytes have so far only been regarded as neuronal supportive cells, assisting with CNS homeostasis regulation (46), various studies indicate astrocytes' role in the regulation of microglial phenotypes and functions, as well as the innate immune response within the CNS (43,47,48), making them secondary targets for EMF therapy of the CNS. One of the main signaling molecules within the astrocyte activation cascade is ORM2, which plays a role in proinflammatory cytokine release. This, together with their ability to restrict the penetration of immune cells through the blood brain barrier (BBB), makes astrocytes active players in neuroinflammation and subsequent neurorestoration (49). Depending on the nature of the stimuli, they can promote tissue regeneration and repair (A2 reactive astrocytosis) or amplify the immune reaction and cause further tissue damage (A1 reactive astrocytosis) (44,50).

Through secretion of ORM2, which blocks CCL4-CCR5 interaction (51), astrocytes can either inhibit microglial activation and proinflammatory cytokine release or enhance microglial activity through up-regulation of LCN2 (52,53), MCP-1/CCL2 (54), IP-10/CXCL10 (54), or TGF-β (55). Moreover, by expressing innate immune pattern recognition receptors (PRRs), such as Toll-like (TLRs), NOD-like (NLRs), complement, mannose, and scavenger receptors, astrocytes establish a close crosstalk with the surrounding microglia within the CNS in order to eliminate pathogens, restore the tissue, or initiate scar formation (43,47,56,57). Furthermore, recent studies have shown that microglia-astrocyte crosstalk is a vital step in the CNS's innate response to inflammation or injury, revealing that the astrocyte's response to TLR2, TLR3, and TLR4 is greatly enhanced by, or directly related to, the presence of microglia within the surrounding tissue (58,59). This indicates a crucial interaction between astrocytes and microglia in neurorestoration and neurorepair and defines them as crucial molecular targets for EMF therapy and crucial players in potential subsequent neurorestoration.

It is only through carefully coordinated interactions of microglia and astrocytes that inflammatory responses can be regulated and resolved. While A1 astrocytes are pro-inflammatory, exhibiting the up-regulation of genes potentially destructive to synapses, and are induced by microglial secretion of Il-1α, TNFα, and C1q, A2 reactive astrocytes secrete proteins that promote CNS synaptogenesis (43). On top of this, since they are activated under ischemic conditions, A2 astrocytes possess neuroprotective and neurorestorative functions and show the phenotype needed to be evoked for EMF therapy to effectively cause neuroregeneration (50,60,61).

Working together with astrocytes, the most abundant cells within the CNS – microglia – usually serve as the astrocyte’s “chaperone,” regulating astrocyte’s innate immune functions under pathological conditions by releasing factors impacting intracellular signal transduction through STATs and MAPK pathways. In order to further amplify the inflammatory reaction, microglia up-regulate the nuclear factor-κB (NF-κB) signaling pathway. Once activated, astrocytes increase proinflammatory gene expression and enhance the production of proinflammatory cytokines, chemokines, and growth factors (62).

In turn, astrocytes modulate microglial functions and phenotypes through astrocyte-derived factors, chemokines, cytokines, complemented proteins, and, most interestingly, calcium ions, which are greatly prone to the influence of the EMF (63).

*The role of ATP, intra- and extracellular Ca^2+^, HSP, and HIF1α in astrocytes and microglia and their crosstalk*. Underlying causes of damage or degeneration within the CNS might differ, but the secondary effects after or during the inflammation response exhibit similar patterns. Secondary injury due to microvascular or metabolic dysfunction causes a spike in glutamate release and subsequent excitotoxicity, mitochondrial dysfunction, overly active production of reactive oxygen species (ROS), and disbalances in ion concentrations (64). Additional extracellular glutamate then activates the N-methyl-D-aspartate receptors within the neurons and allows calcium influx (64,65). The newly established calcium influx causes calcium excitotoxicity, which, in turn, disturbs the mitochondrial function and causes excessive ROS production, ultimately resulting in acute necrotic cell death or delayed apoptotic cell death (65-67).

Another important characteristic of activated astrocytes is the elevated intracellular calcium (Ca^2+^) concentration (68). In order to communicate, astrocytes initiate the transfer of inositol triphosphate (IP3) through gap junctions and extracellular adenosine triphosphate (ATP) signaling. This causes a calcium wave, increasing the Ca^2+^ in all adjacent cells (46,68). This increased Ca^2+^ then contributes to up-regulation of downstream calcium-dependent phosphatases and protein kinases, changing astrocyte's morphology and mobility (69).

Similar to the calcium wave propagating to surrounding microglia, ATP released from astrocytes can also mediate the astrocyte-microglia crosstalk and activate the local microglia due to their high expression of purinergic receptors (70). Purinergic receptors, appearing in P1, P2X, and P2Y classes, play a huge role in cell proliferation, cytokine secretion through mediation of ATP (P2), or adenosine (P1) release. While P2Y and P1 receptors are G-protein coupled, P2X receptors are ligand-gated ion channels greatly distributed among neurons and glial cells within the CNS and PNS. Because of their overt presence within the nervous system, these receptors have been implicated as crucial players in mediating the neuron-to-glia and glia-to-vascular-cells communication, and thus regulating neurogenesis, neurodegeneration, neuroinflammation, and neurorestoration (71). As they are ligand-gated ion channels with high affinity for charged particles, EMFs can, indeed, act by modifying their function.

Astrocytes can also inhibit microglial activity through down-regulation of expression of molecules essential for proinflammatory cytokine production, nitric oxide (NO) production, ROS, and TNFα release (68,72-74).

Thus, astrocytes and microglia both play a dual role in neurodegeneration and neuroinflammation by either furthering the immune response and postponing restoration or decreasing the inflammation and initiating neuroprotection. This makes them potential targets for electromagnetic field therapy of the CNS, enabling initiation of neurorestoration.

## METHODS

### Eligibility criteria

Our systematic review addressed published literature targeting the molecular effect of the EMFs on the brain tissue regeneration mediated by the crosstalk of astrocytes and microglia. Literature search was reduced to the experiments that included application of either magnetic or EMF *in vitro* or *in vivo* on the cells typically present in the brain tissue and active during neuroregeneration. Studies performed on other cell types were not included.

### Information sources

Our search was designed using key words and Boolean operators in congruence with Peer Review of Electronic Search Strategies (PRESS) Checklist (75). Final search text wording was as follows: (electromagnetic fields) AND (astrocyte OR microglia OR microglial OR astrocytic OR regeneration OR restoration) AND (brain).

The identical wording was used in search engines of PubMed, Scopus, and Web of Science, without publishing date or language limit. The reference lists of highly relevant studies were hand-searched in order to identify additional studies to be included.

### Search

Search results from all databases were compared and the duplicates removed. Primary screening resulted in classification of studies as those with relevant, uncertain, and irrelevant status. Secondary screening included accessing the abstract in order to definitely confirm the relevance of the screened studies and decide on the studies previously classified as having uncertain status. All relevant literature was thoroughly studied in order to extract the data and make conclusions about the research question.

## RESULTS

### Study selection

**Figure 1:**
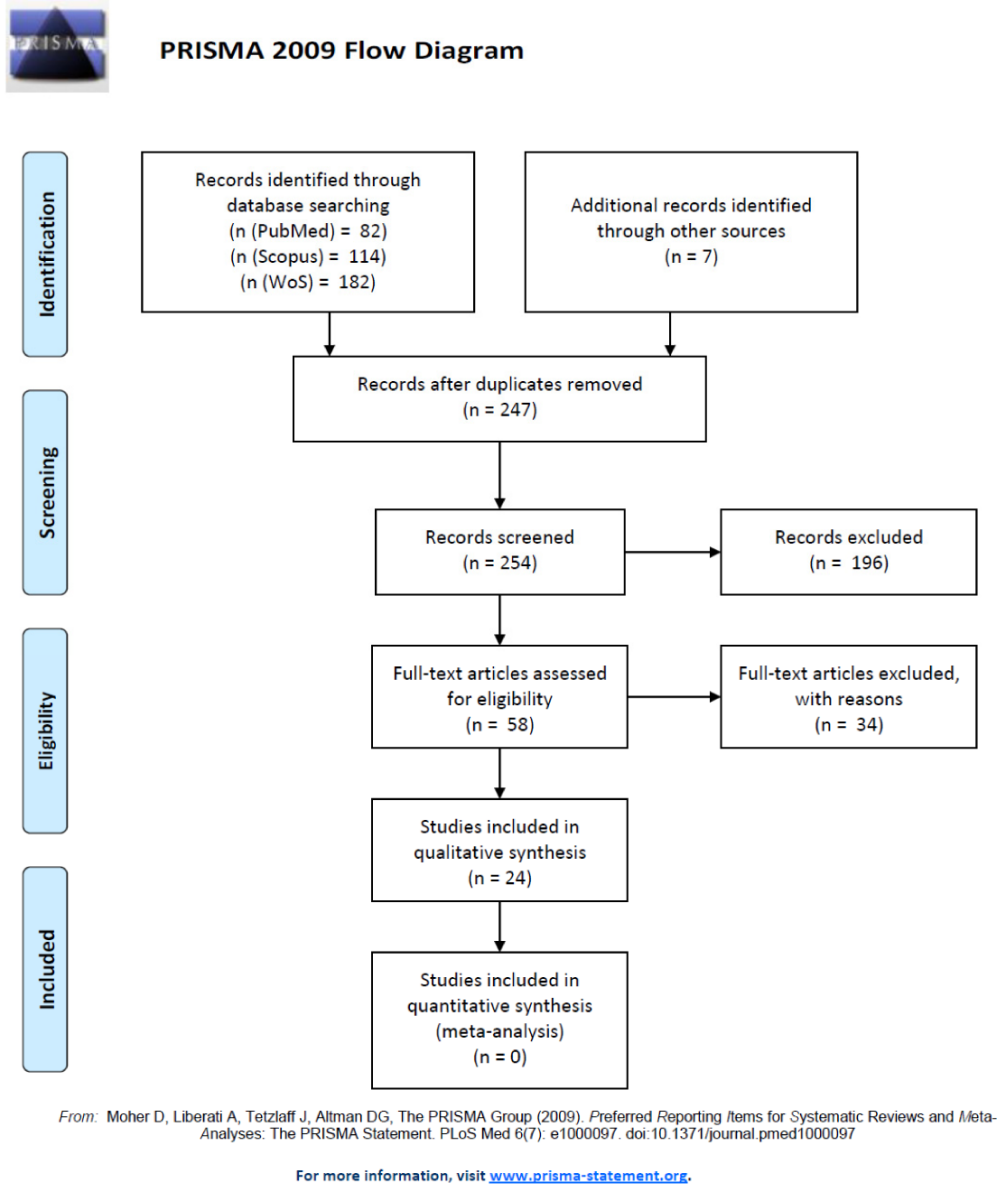
The Preferred Reporting Items for Systematic Reviews and Meta-Analyses (PRISMA) 2009 Flow Diagram delineating our study selection process.

We found 82, 114, and 182 studies in PubMed, Scopus, and Web of Science, respectively. Our manual search identified additional 7 studies that were included in the results list during the search. In the next step, 131 duplicates were removed from the list, leaving 247 articles for primary and secondary screening. The final list of eligible studies consisted of 24 articles addressing our research question in at least one complete textual paragraph. All of the remaining articles were thoroughly read and used in the final synthesis (Figure 1).

*Molecular basis of microglia and astrocyte-mediated neurorestoration upon EMF application.* Based on the current knowledge and the role of astrocyte-microglia crosstalk in neurorestoration, one of the main mechanisms behind the regenerative properties of EMF is its regulation of the microglial and astrocytic activity and, with that, the function of the innate immune system (4-6).

According to Rosado et al (4), the main targets of EMF in innate immunity are HSPs, extracellular ATP, and HIF1α, which are all present within the CNS and play an important role in glial cell functioning (43,68). Besides acting just upon macrophages, HSP, extracellular ATP, and HIF1α, EMF also plays a role in the pathways that activate astrocytes and microglia. This implies that EMF might act within the CNS to initiate neurorestoration and restore proper functioning (4,43,68). Moreover, by investigating the important role of Ca^2+^ in intracellular signaling and astrocyte and microglial crosstalk, EMFs, and microglial and astrocytic activation, a few studies have specifically explored the molecular mechanisms of microglia- and astrocyte-driven neurorestoration (76-78). Therefore, some of the mechanisms at work during EMF-triggered neurorestoration could also be deduced from the existing studies on EMF influence on Ca^2+^ and ATP concentration done on different cell types, and then expanded further to suggest molecular mechanisms of EMF action upon astrocytes and microglia after EMF treatment.

*ATP signaling intra- and extracellular Ca^2+^ concentration, and EMF*. Elevated extracellular ATP concentration is a damage signal that works as a chemoattractant and induces the release of inflammatory cytokines. Within the CNS, ATP is released by both neurons and astrocytes, but under pathological conditions, astrocytes serve as the biggest source of synaptic adenosine (79). In order to mediate its action, there exist four types of G-protein-coupled adenosine receptors (ARs), which can all be found in both astrocytes and microglial cells (80). While A1 and A3 ARs inhibit adenylate cyclase activity and decrease cAMP, A2A and A2B ARs increase cAMP accumulation. Another mechanism of ATP recognition within microglial cells is through the P2X purinoceptor 7 (P2RX_7_). The P2RX_7_ receptors serve as PRRs for extracellular ATP-mediated apoptotic cell death and inflammation (81-84).

Studies assessing the influence of EMF on ATP signaling and intra- and extracellular Ca^2+^ in astrocytes are scarce, have been made with extremely low frequency magnetic fields (ELF-MF) and ELF-EMF, respectively (34,85), and look at *in vitro* astrocyte cultures. However, computational models that studied the influence of EEG field on calcium waves in the neocortex (39) do suggest potential effects of ELF-MF, ELF-EMF, and EEG on ATP signaling and extracellular Ca^2+^ concentration in astrocytes.

While Clarke et al (85) have shown that ELF-MFs increase intracellular calcium levels within the cytoplasm of the cultured astrocytes, Golfert et al (34), by studying microvesicle motility of astrocytes in *in vitro* cultures, have also suggested that ELF-EMFs change calcium levels by increasing calcium influx. Increased Ca^2+^ ion influx is usually a product of astrocytic induction of diffusion of IP3 through gap junctions and extracellular ATP signaling and, in normal conditions, it activates the surrounding microglial cells and changes astrocytes’ morphology and mobility. This is why it presents one of the main molecular targets for EMF therapy of neurodegenerative and neuroimmune disorders.

On the other hand, the computational model by Ingber et al (39) also suggests that macrocolumnar EEG fields can significantly influence Ca^2+^ momentum waves, increasing their frequency and occurrence. Although EEG has not so far been considered a method of EMF therapy for neurorestoration, this computational model suggests that, in its altered form, it could potentially be used as a method for initiation of neurorestoration.

*HIF1α and EMF.* HIF1α is a transcription factor that plays a role in activating microglia in ischemic stroke and tissue damage. It is involved in microvascular dysfunction by recruitment of T-lymphocytes and in tissue damage by activation of innate inflammation. Within the CNS, HIF1α is mainly expressed by glial cells and neurons (86) and its overexpression contributes to tissue damage in the nervous system. Multiple studies have shown that localized application of EMF inhibits HIF1α through decreased release of interleukin (IL-1β), TNF-α, IL-6, IL-8, and human monocyte chemoattractant protein-1 (MCP-1/CCL2). As IL-1β, TNF-α, IL-6, IL-8, and MCP-1/CCL2 serve as major pro-inflammatory signals, their down-regulation significantly reduces the inflammatory response, cell death, and apoptosis (31,87). As the inflammatory response plays a huge role in tissue damage, down-regulation of HIF1α through EMF activity might be one of the mechanisms behind the neurorestorative processes within the CNS.

An *in vitro* study by Huang et al (88) has shown that overexpression of HIF1α inactivates microglia during re-exposure to hypoxia and reduces their activity and could, therefore, be a novel method of achieving neuroprotection after ischemic stroke or brain ischemia by diminishing the possibility of occurrence of A1 reactive astrocytosis. Even though this study has not applied EMF, and the reported effects of HIF1α are indeed contradictory to the accepted role of HIF1α in anti-inflammatory processes, it suggests that HIF1α overexpression might have neuroprotective effects, especially when it comes to utilizing intermittent hypoxia for protecting the CNS from ischemic damage. This effect should be explored further using EMF application. On the other hand, Vincenzi et al (31) have shown, in their study of PEMF influence on microglia, that down-regulation of HIF1α has anti-inflammatory effects and that the application of PEMF inhibited HIF1α activation. As their results contradict the function of HIF1α demonstrated by Huang et al (88), more studies need to be done on the activity of HIF1α in microglia after EMF application in order to further elucidate its role in the induction of A1 or A2 reactive astrocytosis and neurorestoration.

*HSP and EMF.* Heat shock proteins are molecular chaperones involved in protein folding and activation of antigen-presenting cells (APCs), and induce the adaptive immune response by promoting the secretion of inflammatory cytokines in APCs. It has been shown that ELF-MFs rapidly induce the expression of HSP in mouse macrophages and human leukemia cells (4). This reaction causes ROS expression and scavenger inhibition of the free radical production and expression of HSP70. When it comes to just the intracellular HSP70 concentration, multiple studies have been done showing that HSP70 can decrease the signaling of proinflammatory factors (NFκB, MMPs, and ROS) after EMF application in human K562 cells and HL60 cells in vitro (89,90).

Although none of the studies mentioned above looked at HSP expression in astrocytes or microglia, Bodega et al and Watilliaux et al have shown no changes in HSPs expression in astrocytes after MF and EMF application, respectively. While Bodega et al (91), in an *in vitro* study of astrocytes, have shown that exposure to a static magnetic field does not change the concentration of HSP25, HSP60, and HSP70, Watilliaux et al (92) have, in an *in vivo* study on the brain of developing rats, demonstrated that EMF application does not change the expression and abundance of HSP60, HSP70, and HSP90.

Since none of these studies differentiated between intracellular and extracellular HSP, and some studies in astrocytes have shown no change in HSP expression after EMF application, this phenomenon needs to be further researched. The effect of EMF upon HSP expression with the purpose of initiating neurorestoration needs additional clarification.

## DISCUSSION

Due to the microglia-astrocyte crosstalk, application of EMF on microglia not only impacts their function and mobility, but it also dictates the behavior of the surrounding astrocytes and their response within the tissue.

Although the studies mentioned in our review made many valuable conclusions specifically outlined above, here we want to additionally stress our interpretation of the observed data and bring forth some further hypotheses. On top of working together with astrocytes to initiate the immune response within the CNS, microglia also play a crucial role in determining the fate of astrocytes – inducing either A1 or A2 reactive astrocytosis. Once classically activated, microglia then secrete Il-1α, TNFα, and C1q and induce A1 astrocytic phenotype. Induced astrocytes, without their ability to promote growth and survival of neurons and synaptogenesis, cause neuronal and oligodendrocyte’s death and further degeneration. In order for microglia to have neuroprotective and neuroregenerative effects they must induce A2 reactive astrocytosis through down-regulation of Il-1α, TNFα, and C1q expression. As Vincenzi et al (31) have shown that EMFs act upon microglia by reducing the expression of HIF1α, which is caused by the decrease in Il-1α and TNFα concentration, it is plausible that this reduction in Il-1α, TNFα, and HIF1α induces A2 reactive astrocytosis.

As much as astrocytes play an indispensable role in physiological conditions and the immune response within the CNS, they also release neurotrophic factors such as transforming growth factor beta (TGF-β) and nerve growth factor (NGF), which play a role in the formation of the glial scar. Even though it was previously thought that the glial scar hinders axonal regeneration, recent studies have shown that the ablation of chronic astrocytic scars disabled the spontaneous regrowth of transected axons in spinal cord injury (SCI) lesions and increased axonal dieback (93). Thus, contrary to the accepted dogma, the formation of an astrocytic scar is crucial to axon regrowth and is a pivotal step in neurorestoration (93,94). Molecular triggers that lead to this scar formation include epidermal growth factor, fibroblast growth factor, endothelin-1, and ATP. By repairing the BBB, decreasing the rate of neuronal degeneration, and reducing the infiltration of inflammatory cells into the CNS, astrocytic scar thus aids the recovery of function and prevents further functional deterioration (94). Interestingly, in most cases activated microglia will drive the astrocytes to the harmful, A1 reactive astrocytosis, but once triggered by the application of EMF, the normal functioning of microglia is disrupted through Ca^2+^ influxes, subsequent excitotoxicity, oxidative stress, and apoptosis. This disrupted functioning decreases the expression of Il-1α, TNFα, and HIF1α, which changes the nature of interaction between astrocytes and microglia, possibly drives astrocytes toward A2 reactive astrocytosis, and initiates neurorestoration. Here, we assume that, since overexpression of Il-1α, TNFα, and HIF1α induces A1 reactive astrocytosis, their decreased expression could induce A2 reactive astrocytosis, which up-regulates many neurotrophic factors and should have a protective function (45).

Thus, one of the key mechanisms used by astrocytes and microglial cells for communication is ATP- and glutamate-mediated calcium signaling evoked by the propagation of an action potential. Triggering the axonal release of ATP, this mechanism acts as a key player in mediating cell-to-cell communication within the CNS and enables the recruitment of new cells to the injury site if there is a need for an inflammatory response. It is this astrocytic ATP release that sets off the intracellular Ca^2+^ waves and Ca^2+^ propagation among neighboring cells, triggering microglial Ca^2+^ response as well via P2RX_7_ receptors. One of the major features of astrocytes is that they release ATP upon mechanical or electrical stimulation or glutamatergic receptor activation (95,96). When stimulated by EMF, astrocytes decrease the secretion of the extracellular ATP concentration, dampening the pace of the microglial response and preventing further tissue damage (97). The exact mechanism behind this action remains to be uncovered.

Looking at ATP concentration and signal processing upon EMF application, an *in vitro* study by Ongaro et al (98) has also shown that EMFs up-regulate A2A and A3 ARs in human osteoarthritic synovial fibroblasts (HOSF), thus decreasing extracellular Ca^2+^ . If this mechanism of action were to be detected in astrocytes as well, it would suggest that the decreased Ca^2+^ diminish the occurrence of calcium waves, whose reduced occurrence down-regulates all the phosphatases and protein kinases, changing astrocyte's morphology and mobility (68). Coupled with a lower extracellular ATP concentration, this should then decrease the extent of the immune reaction. Although this study was done on HOSF, based on the properties of ARs present in astrocytes, it still presented valuable results suggesting a novel potential molecular mechanism behind the action of EMFs on astrocytes and could be repeated for astrocytes.

**Figure 2:**
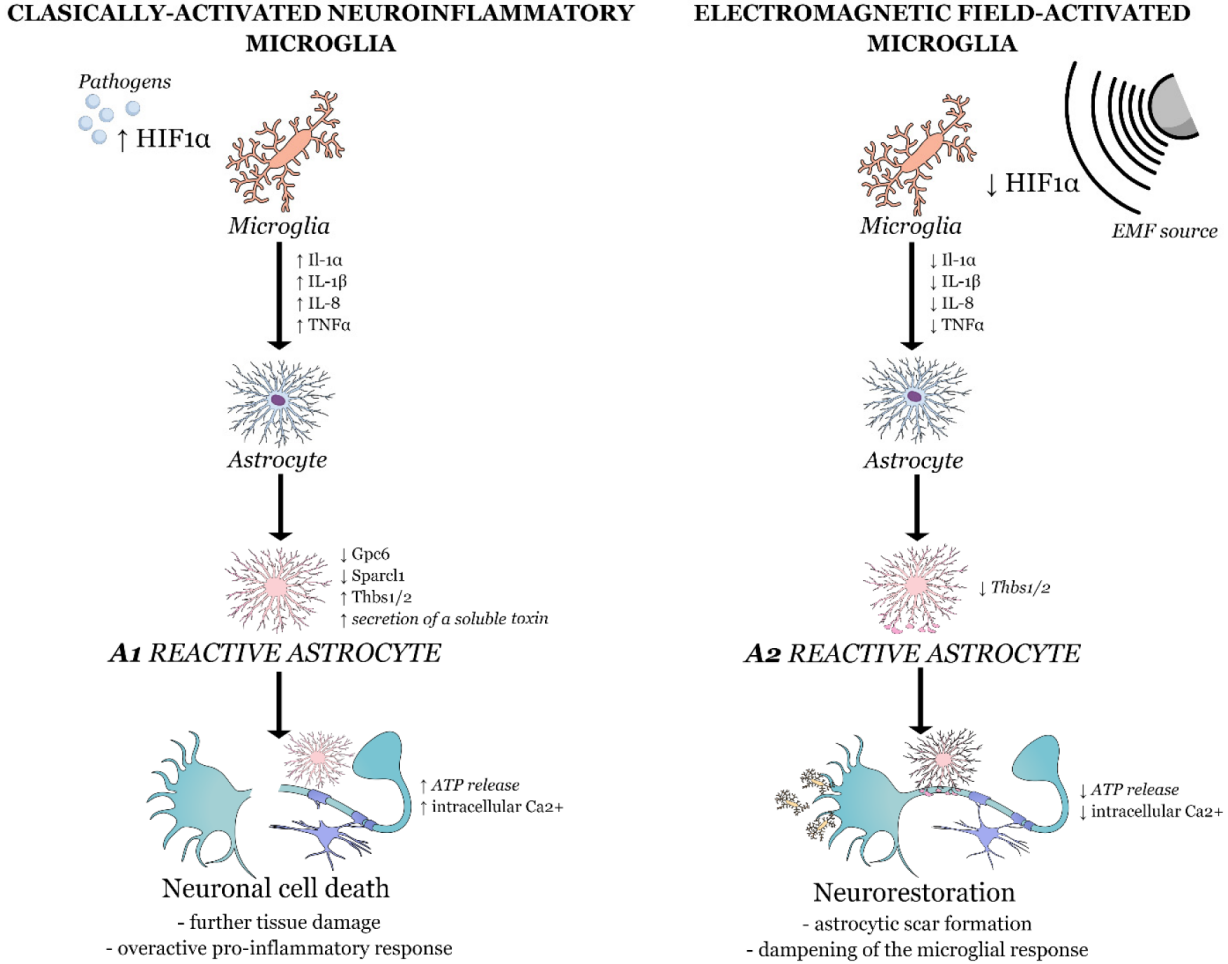
Hypothesized microglia-astrocyte crosstalk after electromagnetic field (EMF) application and its role in neurorestoration (*Source:* Mind the Graph on *https://mindthegraph.com/,* used under the CC BY-SA license)*.*

Ongaro et al (98) have also shown that, through their action upon A2A and A3ARs, EMFs down-regulate the expression of TNFα and IL8, which, as in the case with the down-regulation of the HSP cascade, reduces inflammation – as it was suggested by Vincenzi et al (31). While TNFα functions as a systemic inflammation cytokine and regulates the immune cell response, IL-8 is a chemokine produced mainly by macrophages within the CNS and serves as a chemoattractant, recruiting neutrophils to the site of damage or infection. This decrease in the rate of the immune reaction enables glial cells to recruit other cell types to the injury site and initiate neurorestoration (93). A graphical representation of our hypotheses on the molecular mechanisms behind the microglia-astrocyte crosstalk post-EMF application can be found in Figure 2.

Looking at further *in vitro* studies relating EMF application with ATP and ROS levels, Feng et al (99) have shown that ELF-MF application induced mitochondrial permeability transition through increase of intracellular ROS levels in human amniotic (FL) cells. On the other hand, Destefanis et al (100) have shown that the application of EMF increased mitochondrial activity through transcriptional modulation of mitochondrial respiratory complexes without any noticeable change in ATP levels. As the study by Destefanis et al contradicts some of the other research in the field suggesting EMFs role in decreasing extracellular ATP levels, more experiments must be done to elucidate this issue.

Finally, when it comes to HSP70 expression after EMF application in human leukemia cells and mouse macrophages, which show an increase in concentration of HSP70 (4), the studies by Bodega et al (91) and Watilliaux et al (92) on *in vitro* and *in vivo* astrocytes, respectively, show no changes in HSP70 expression following EMF treatment. As Bodega et al have used an *in vitro* culture and Watilliaux et al have used the brain of developing rats, the results of both studies could not be reliable or applicable to adult rat brains or the human brain, so more studies need to be done on this problem to definitely conclude that EMF has no effect on HSP70. Acting upon NF-κB, expression of HSP70 causes immunosuppression and inhibits apoptosis and, within the CNS, could, upon application of EMF, halt the immune response long enough for the glial cells to initiate the restoration of the neuron’s structure and function.

## Perspectives for future research

Although significant progress has been made in delineating the molecular mechanisms of action of astrocytes and microglia after EMF application, a considerable work still lies ahead. In order to pinpoint the exact mechanisms at work during proposed EMF-initiated neurorestoration further research is needed on EMF’s influence on Ca^2+^ levels in both astrocytes and microglia, ideally both *in vivo* and *in vitro*. As the immune response of astrocytes and microglia, and their drive to regenerate the tissue or cause further tissue damage, depends highly on their successful crosstalk, all the further research, if done in *in vitro* cultures, should be performed in co-cultures of astrocytes and microglia.

Moreover, as opposed to just observing the HSP levels in short time spans after EMF treatment, the EMF should be applied in variable time intervals and at various intensities. As Bodega et al (91) have only looked at the influence of static sinusoidal (50 Hz) and combined static/sinusoidal magnetic fields on astroglia in culture 24 h after exposure, a study with longer exposure times (over 48 h) and with additional pulsed magnetic fields, and potentially dynamic EMFs, should be performed. As opposed to the field with a static flux density, the flux density of pulsed magnetic fields changes at various predetermined frequencies could, thus, evoke a higher rate of response and a stronger response from exposed cells than pure static fields. If the cells were exposed to static fields for longer periods of time, the field strength would raise the cell’s threshold and it would no longer have any influence on the cell’s function, migration, or action. This could be one of the reasons why Bodega et al (91) have observed no difference in HSP expression after MF application. On the other hand, Watilliaux et al (92) have used a higher frequency, 1800 MHz pulsed EMF for 576 s on the brain of developing rats at post-neonatal days 5, 15, or 35. Although their study used PEMF, the limiting factors, which were also the factors the authors set to explore, were the age of the rats and the short EMF application time. As this was a study aiming to explore the adverse effects of mobile phone use on children, another study aiming to specifically observe the effects of PEMF on adult brain could be repeated with the same setup on adult rat brains, but with longer EMF exposure times, and varying field intensities to study whether EMFs, in any form, could affect HSP expression.

When it comes to HIF1α’s role in neuroregeneration, Vincenzi et al (31) and Huang et al (88) presented opposite results. Therefore, a study similar to that by Vincenzi et al looking at pro-inflammatory cytokine expression in microglia, HIF1α concentrations, and ROS levels needs to be done with longer PEMF exposure times (over 48 h of incubation) and alternating PEMF strengths to evaluate the optimal strength needed to inhibit HIF1α expression and to confirm the beneficiary effects of PEMF on neurorestoration. When it comes to the study by Huang et al (88), as instrumental as it is to the recognition of the positive role HIF1α in neurorestoration, it should be repeated with the same setup but with the addition of PEMF generators.

Finally, looking at the influence of EMF therapy on Ca^2+^ and ATP concentrations in microglia and astrocytes, additional studies could be done in co-cultures of astrocytes and microglia to quantify the EMF influence on HSP levels, and with PEMF and ELF-EMF to study the differential effects of specific field patterns on Ca^2+^ and ATP concentrations.

## Conclusion

Observing the effects of post-EMF therapy, when microglia get activated through voltage gated ion channels, multiple studies have reported a decrease in inflammatory response following subsequent astrocyte recruitment, which, together with microglia, enable astrocyte scar formation – ultimately aiding neurorestoration and axonal regeneration (1-8,93).

On the other hand, if the inflammatory response within the CNS is weak or lacking, the EMF therapy could play a crucial role in its proper activation and regulation though immunosuppression and inhibition of apoptosis. When it comes to neurorestoratory A2 reactive astrocytes, their pathway highly depends upon the microglia-astrocyte interaction involving the down-regulation of astrocytic P2Y1 purinergic receptors and formation of an astrocytic scar (43,93,94) and is, to an extent, prone to the influence of EMF (28,31,34,98). When it comes to the influence of EMF on the scar-formation, its onset is induced by the change in the microglia-astrocyte crosstalk through up-regulation of extracellular ATP, which activates the A2 reactive astrocytosis pathway, initiates the release of anti-inflammatory cytokines, and speeds up its formation.

Having said all this, there is still more to uncover when it comes to the impact of EMF on the microglia-astrocyte crosstalk and neurorestoration, especially considering that some studies regarding EMF’s impact on ATP and HSP concentrations are contradicting. However, the field is advancing in the right direction – suggesting future possible uses of EMF in aiding neurorestoration and initiating neuroprotection.
